# Supplementary figures and images for: The antisecretory peptide AF-16 may modulate tissue edema but not inflammation in experimental peritonitis induced sepsis
Source: PLoS One. 2020 Aug 21;15(8):e0232302. doi: 10.1371/journal.pone.0232302 (PMC7446908; doi:10.1371/journal.pone.0232302)

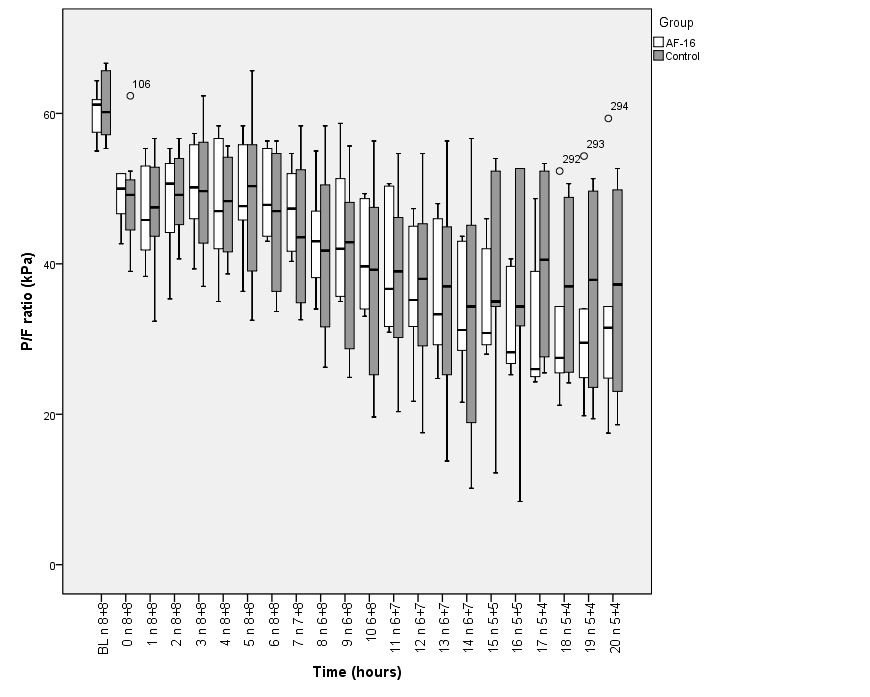

Supplement: S1 Appendix — Decrease in PaO2/FIO2 ratio from baseline and throughout the 20 hours observation period in both groups. Reported on an hourly basis. (TIF) [file pone.0232302.s001.tif]

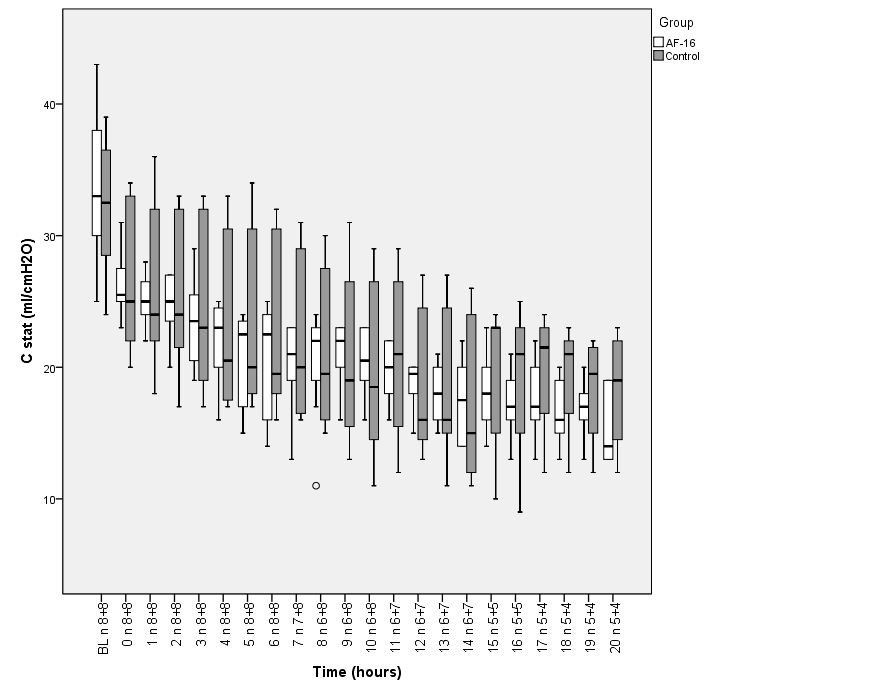

Supplement: S2 Appendix — Static compliance (ml/cm H2O) measured every hour of the twenty hours observation period. Decrease in compliance in both intervention and control groups. (TIF) [file pone.0232302.s002.tif]

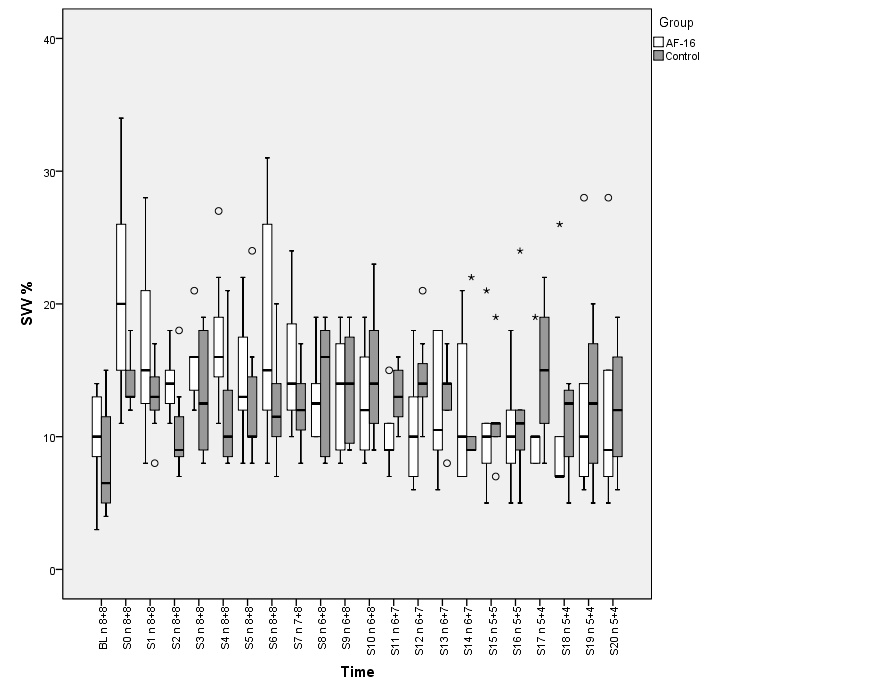

Supplement: S3 Appendix — SVV (%) monitored continuously at the bedside and reported on an hourly basis during the 20 hours observation period in both groups. (TIF) [file pone.0232302.s003.tif]

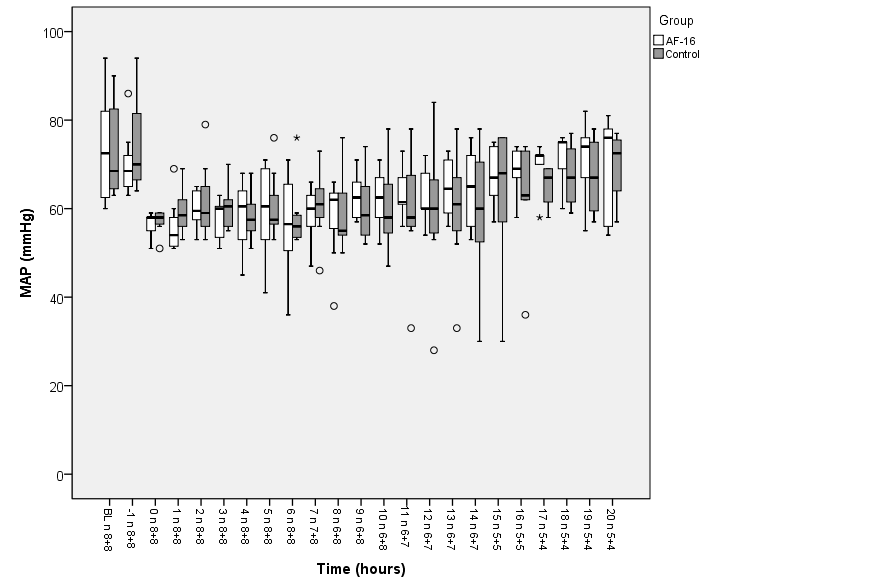

Supplement: S4 Appendix — MAP (mmHg) measured continuously at the bedside and recorded on an hourly basis in both groups during the 20 hours observation period. (TIF) [file pone.0232302.s004.tif]

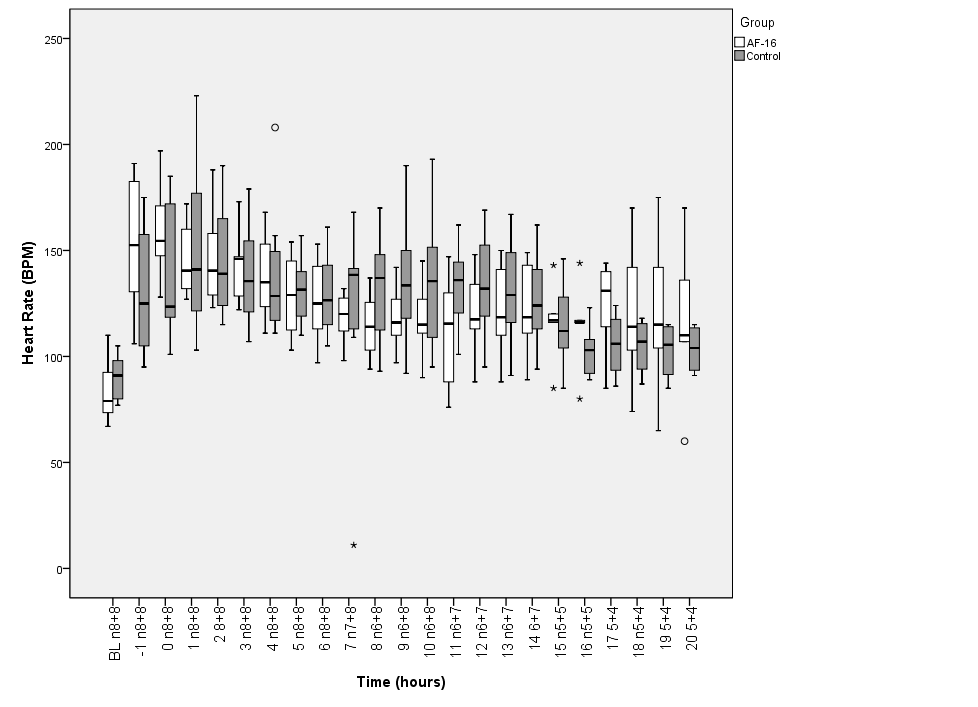

Supplement: S5 Appendix — Evolution of heart rate (beats per minute) at an hourly basis during the twenty hours observation period, shows an increase in heart rate in both groups. (TIF) [file pone.0232302.s005.tif]

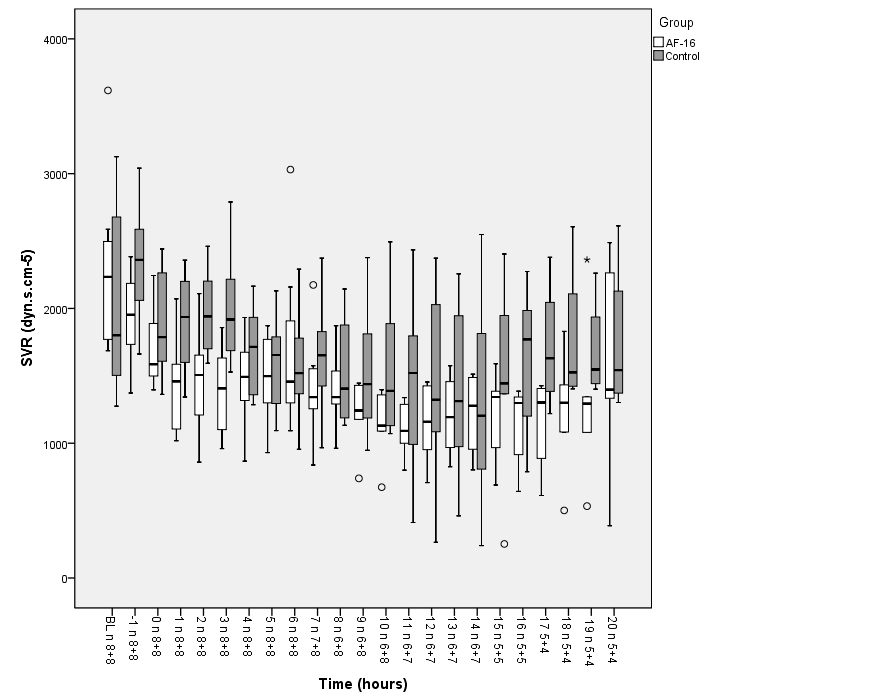

Supplement: S6 Appendix — Systemic vascular resistance (SVR) calculated for intervention and control groups, respectively, on an hourly basis during the 20 hours observation period. (TIF) [file pone.0232302.s006.tif]

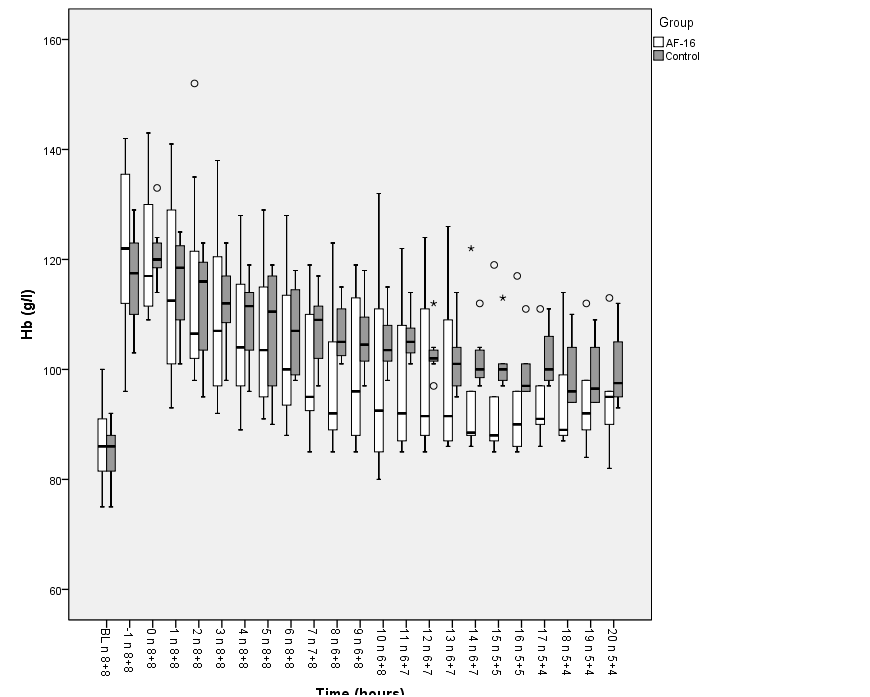

Supplement: S7 Appendix — Hemoglobin concentration (g/l) measured every hour in both groups during the 20 hours observation period. (TIF) [file pone.0232302.s007.tif]

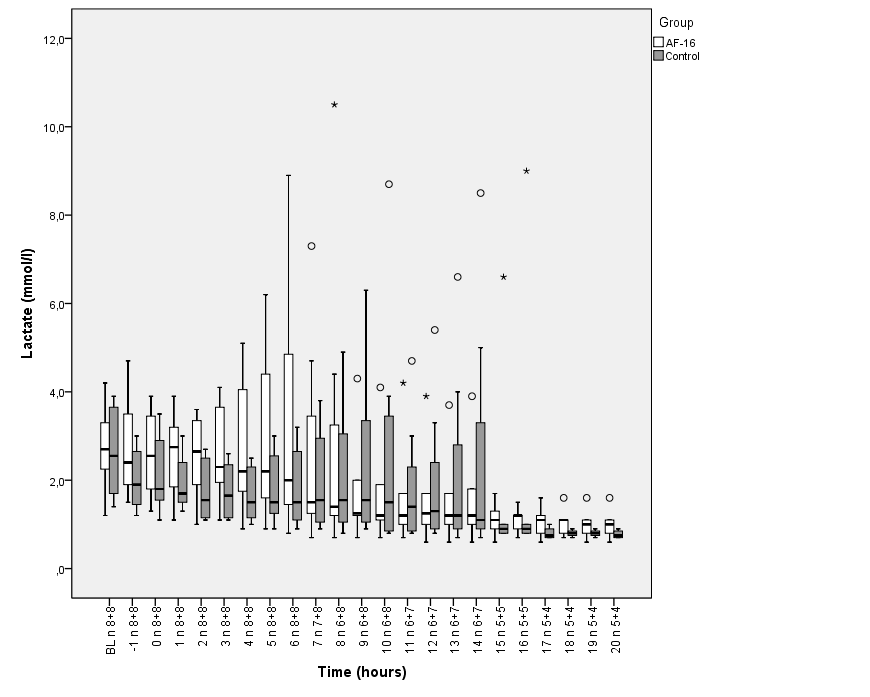

Supplement: S8 Appendix — Arterial blood lactate concentration (mmol/l) measured every hour in both groups during the 20 hours observation period. (TIF) [file pone.0232302.s008.tif]

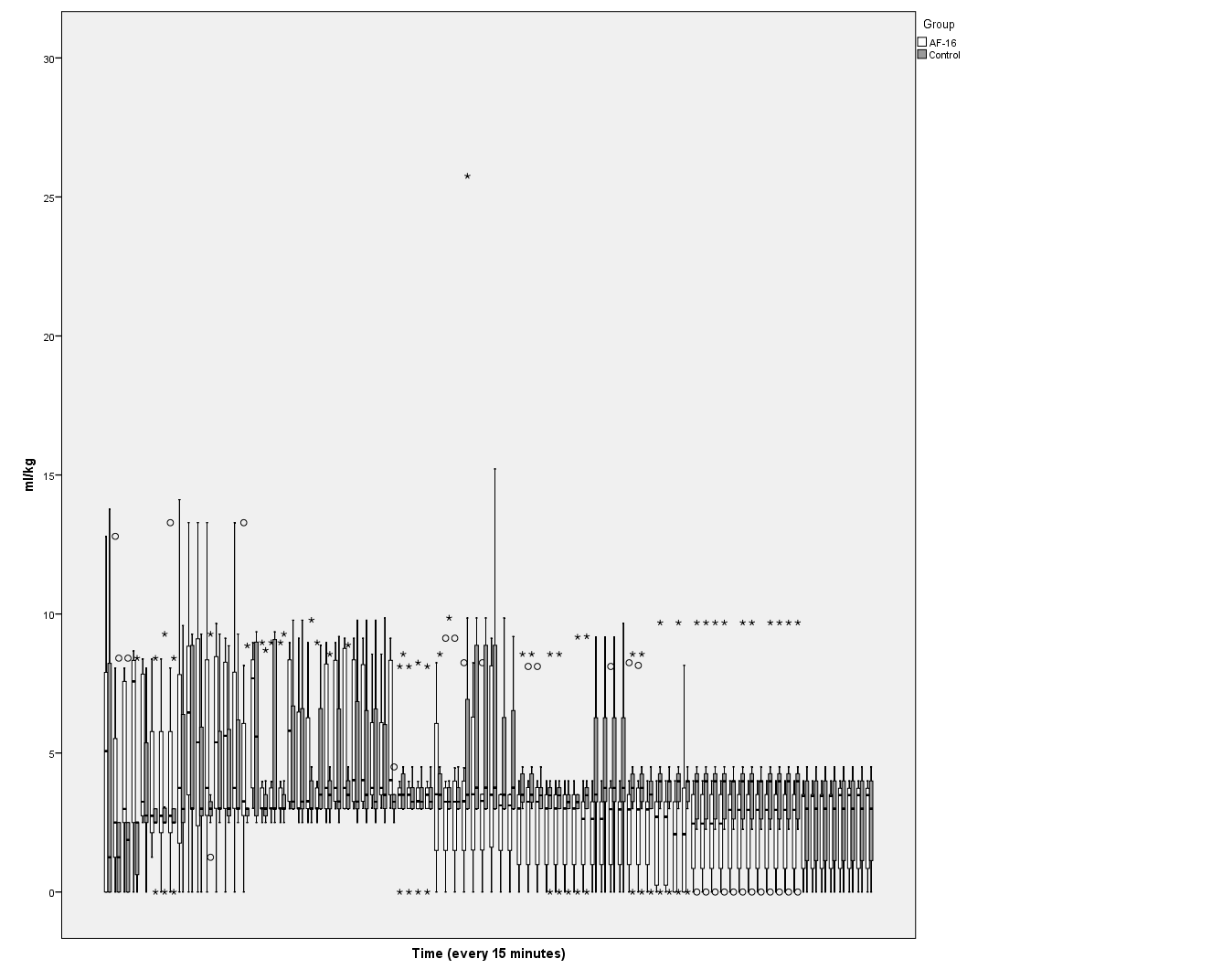

Supplement: S9 Appendix — Total fluid requirements during resuscitation period of maximum 20 hours, reported in ml/kg every 15 minutes. (TIF) [file pone.0232302.s009.tif]

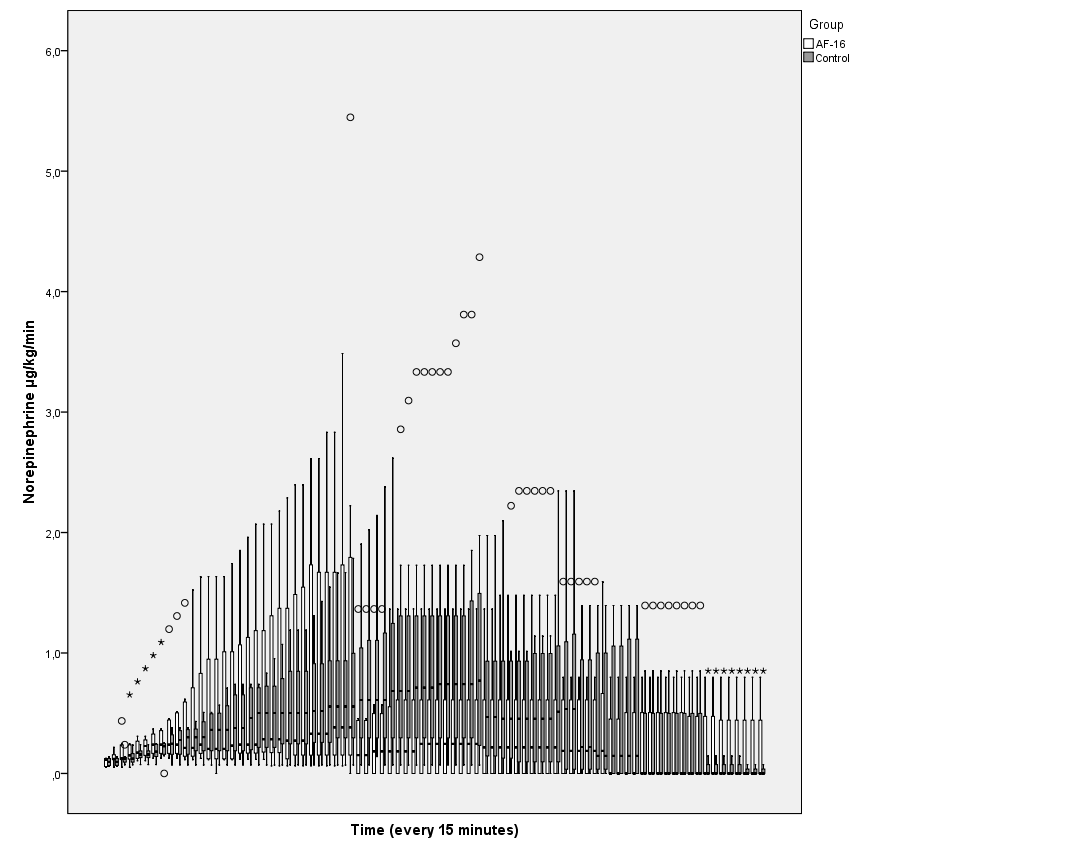

Supplement: S10 Appendix — Norepinephrine consumption in μg/kg/min registered continuously and reported every 15 minutes during the observation period. (TIF) [file pone.0232302.s010.tif]
