# Supplementary material for: The antisecretory peptide AF-16 may modulate tissue edema but not inflammation in experimental peritonitis induced sepsis
Source: PLoS One. 2020 Aug 21;15(8):e0232302. doi: 10.1371/journal.pone.0232302 (PMC7446908; doi:10.1371/journal.pone.0232302)
Supplement: S11 Appendix — Peritonitis induced sepsis in pilot study (four pigs) to study model, pig 4* received intervention (AF-16). (DOCX) [file pone.0232302.s011.docx]

**S11 Appendix. Pre-study peritonitis model.**

|  | **Peritonitis before sepsis (h)** | **Sepsis (h)** | **Noradrenalin**  **µg/kg/h of sepsis** | **Fluid**  **ml/kg/h of sepsis** | **Diuresis**  **ml/kg/h of sepsis** | **Fluid total**  **ml/kg/h**  **of sepsis** |
| --- | --- | --- | --- | --- | --- | --- |
| **Pig 1** | 4 | 8 | 80 | 14 | 0,4 | +14 |
| **Pig 2** | 4 | 9 | 38 | 14 | 0,3 | +14 |
| **Pig 3** | 4 | 17 | 43 | 15 | 1,2 | +14 |
| **Pig 4*** | 5 | 12 | 93 | 20 | 0,9 | +19 |

**S9 Appendix.** Peritonitis induced sepsis in pre-study (four pigs) to study model, pig 4* received intervention (AF-16).
